# Coronary-Cameral Fistula, Thebesian Veins, and Anomalous Coronary Vein on Cardiac Computed Tomography

**DOI:** 10.7759/cureus.15589

**Published:** 2021-06-11

**Authors:** Jack Xu, Munthir Mansour, Tarun Pandey, Kedar Jambhekar, Subhi J Al'Aref

**Affiliations:** 1 Cardiovascular Medicine, University of Arkansas for Medical Sciences, Little Rock, USA; 2 Internal Medicine, University of Arkansas for Medical Sciences, Little Rock, USA; 3 Radiology, University of Arkansas for Medical Sciences, Little Rock, USA; 4 Cardiology, University of Arkansas for Medical Sciences, Little Rock, USA

**Keywords:** anomalous coronary vein, cardiac ct, coronary anatomy, coronary-cameral fistula, thebesian veins

## Abstract

A 63-year-old female with a history of hypertension presented with progressively worsening shortness of breath. She underwent a cardiac catheterization and was found to have prominent Thebesian veins as well as anomalous connections between the proximal right coronary artery and the right ventricle. Cardiac computed tomography was ordered for further evaluation and showed a coronary fistula to the right ventricular outflow tract confirming the diagnosis of a coronary-cameral fistula (CCF). CCF are rare congenital anomalous communications that occur between coronary arteries and a cardiac chamber. They are usually an incidental finding and patients are rarely symptomatic. As the use of coronary computed tomography angiography is rapidly expanding, the detection of CCF will likely increase in the general population.

## Introduction

Coronary artery anomalies include various congenital disorders whose manifestations and pathophysiological mechanisms can be highly variable. Coronary-cameral fistulae (CCF) are congenital or acquired anomalous communications that occur between coronary arteries and a cardiac chamber [1,2]. Acquired CCF are usually associated with trauma or prior cardiac surgeries. Congenital CCF are usually more common than acquired CCF. CCF are an incidental finding and patients rarely have consequences from them. Here, we present a patient with shortness of breath who was found to have CCF during her evaluation.

## Case presentation

A 63-year-old female with a history of hypertension presented with progressively worsening shortness of breath and chest pain. She was hypertensive and hypoxic on admission, initially requiring noninvasive positive pressure ventilation to support her oxygenation. On physical examination, she was volume overloaded with crackles auscultated on lung examination. On chest X-ray, she had pulmonary edema and was also found to have deeply inverted T waves in the anteroseptal leads on electrocardiography with troponin I of 0.50 ng/mL.

**Figure 1:**
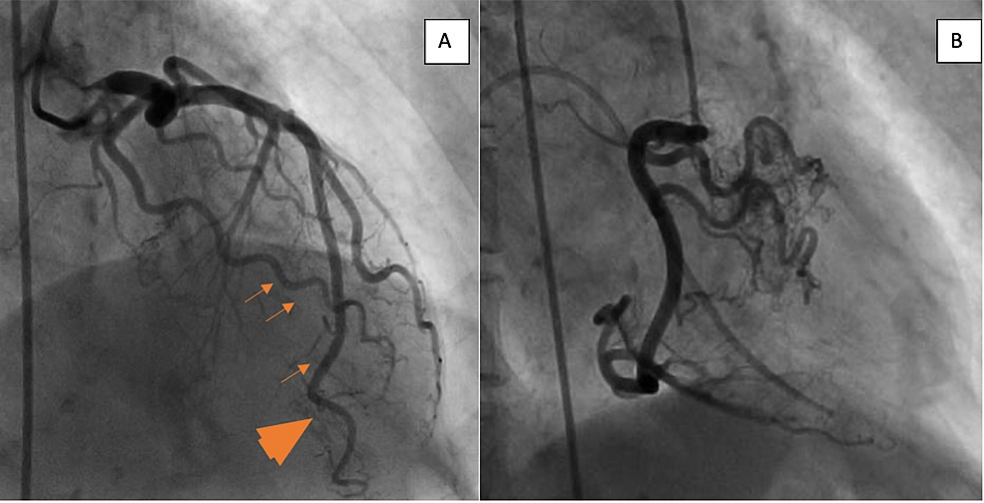
Coronary angiography images. (A) Invasive coronary angiography showing prominent Thebesian veins (orange arrows) with partial opacification of the left ventricular cavity (orange arrowheads). (B) The right coronary artery, in the right anterior oblique projection, showing anomalous connections between the proximal vessel and the right ventricle (note Swan-Ganz catheter in the right ventricular cavity).

**Figure 2:**
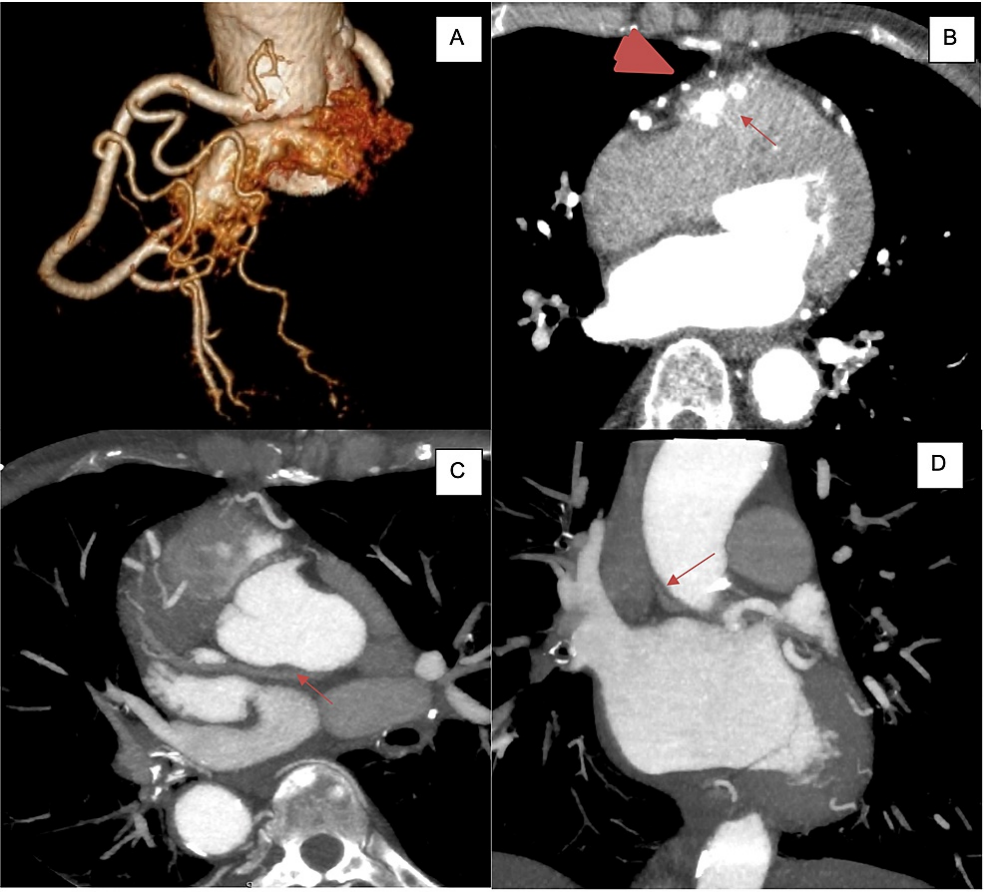
Cardiac computed tomography images. (A) Three-dimensional rendered cardiac computed tomographic image showing a coronary-cameral fistula (part of the right ventricular outflow tract is shown for depiction). (B) Cardiac computed tomography imaging in the axial plane showing multiple arterial branches off the right coronary artery (RCA) (arrowhead) draining into the right ventricular outflow tract (arrow). (C) Axial reformatted computed tomography revealing the course of anomalous coronary vein (originating from the confluence of arterial branches off the RCA and coursing posterior to the aorta). (D) Coronal reformatted images showing the drainage of the anomalous coronary vein into the superior vena cava.

Transthoracic echocardiogram showed a normal ejection fraction with no wall motion abnormalities and an indeterminate diastolic function. A coronary angiogram showed a right dominant system and no significant atherosclerotic coronary artery disease. However, it showed prominent Thebesian veins (Figure 1A) as well as anomalous connections between the proximal right coronary artery (RCA) and the right ventricle (Figure 1B). Cardiac computed tomography (CT) showed a coronary fistula to the right ventricular outflow tract confirming the diagnosis of a CCF (Figures 2A, 2B). An anomalous coronary vein draining the confluence of CCF to the superior vena cava (Figures 2C, 2D) was also seen on the initial arterial phase as well as the delayed venous phase scan. Cardiac magnetic resonance imaging did not show evidence of delayed enhancement in the RCA distribution.

Her symptoms eventually resolved with diuresis along with better control of her blood pressure and she was eventually discharged.

## Discussion

CCF are rare congenital anomalous communications that occur between coronary arteries and a cardiac chamber, but they can also be seen with left or both coronary artery systems [3]. They are most commonly associated with the right coronary system (approximately 55%) and usually drain to the right side of the heart. They rarely terminate into the left ventricle or pericardium. Fistulae are classified as either arterioluminal (direct communication with the cardiac chamber) or arteriosinusoidal (communication via the sinusoidal network) [2]. Historically, they were usually diagnosed via coronary angiogram, but with the advances in noninvasive cardiac imaging, they are being increasingly seen in coronary CT imaging [4,5]. They are usually hemodynamically insignificant but large hemodynamically significant fistulae may cause angina via the coronary steal phenomenon. Fistula-related complications are rare, but they have been associated with myocardial infarction, infective endocarditis, congestive heart failure, arrhythmias, aneurysms, and coronary vessel rupture.

Although there is no consensus on the management of symptomatic fistulae due to their rarity, various techniques have been successfully used including surgical repair, catheter closure, and medical management. Numerous percutaneous catheter techniques have been used including coils, detachable balloons, Amplatzer vascular plugs, and Amplatzer duct occluders. Surgical methods of closure are usually associated with low mortality and morbidity. The risks of fistulae closure include myocardial infarction and migration of coils to either extracoronary or intracoronary structures [2].

## Conclusions

CCF are usually an incidental finding and patients are rarely symptomatic. As the use of coronary computed tomography angiography is rapidly expanding, the detection of CCF will likely increase in the general population.
